# Pleomorphic adenoma of minor salivary gland with therapeutic misadventure: a rare case report

**DOI:** 10.1186/1472-6815-10-2

**Published:** 2010-01-08

**Authors:** Jagdeep S Thakur, Narinder K Mohindroo, Shobha Mohindroo, Dev R Sharma, Anamika Thakur

**Affiliations:** 1 Department of Otolaryngology - Head & Neck Surgery, I. G. Medical College, Shimla, HP, 171001, India; 2 Department of Pathology, I. G. Medical College, Shimla, HP, 171001, India; 3 Department of Pharmacology, I. G. Medical College, Shimla, HP, 171001, India

## Abstract

**Background:**

The benign tumors of nasopharynx are least encountered tumors in otolaryngology, as nasopharynx is considered one of notorious anatomical site for the malignant tumors. Pleomorphic adenoma of the minor salivary gland of nasopharynx and parapharyngeal space is rare. We present a pleomorphic adenoma of minor salivary gland which was mismanaged.

**Case presentation:**

An adult male presented with left nostril obstruction for five months. The examination found big mass extending from nasopharynx to oropharynx. On CT scan, this tumor was quite big and extending to the parapharyngeal space. The FNAB found it a carcinoma but it did not respond to radiotherapy. The excision biopsy of tumor revealed it as pleomorphic adenoma. We found only five published reports on this tumor arising from nasopharynx.

**Discussion and conclusion:**

Although, in this case report exact origin of the tumor could not be ascertained as it also appeared to be a parapharyngeal tumor but we kept the possibility of a nasopharyngeal tumor on the basis of clinical features. The pleomorphic adenoma of nasopharynx is rare. It can be misdiagnosed as malignant epithelial tumor on histopathology. The differentiation from its malignant variant is also difficult. A possibility of benign tumor should always be kept in nasopharyngeal growth with no evidence of metastasis, and histopathological diagnosis of growth should be available before any definitive treatment.

## Background

Benign tumors of nasopharynx are rare since nasopharynx is a common site for malignant tumors. Pleomorphic adenoma is the most common benign tumor of the major and minor salivary glands, but rarely found in the nasopharynx. In this case report, we present a case of a large pleomorphic adenoma arising in the nasopharynx and extending to oropharynx that was diagnosed and managed as squamous cell carcinoma.

## Case presentation

In Sep 2007, a 35-year-old male presented with a progressive nasal obstruction associated with left aural fullness, hearing loss and hyponasal speech for the past five months. Later, the patient saw a mass pushing the soft palate downwards and began having difficulty in swallowing solid food. On examination, there was a mass behind and above the soft palate, which appeared to be arising from nasopharynx. Neck examination was found normal. Past history included a pyelolithotomy and gastrojujenostomy for duodenal ulcer.

**Figure 1:**
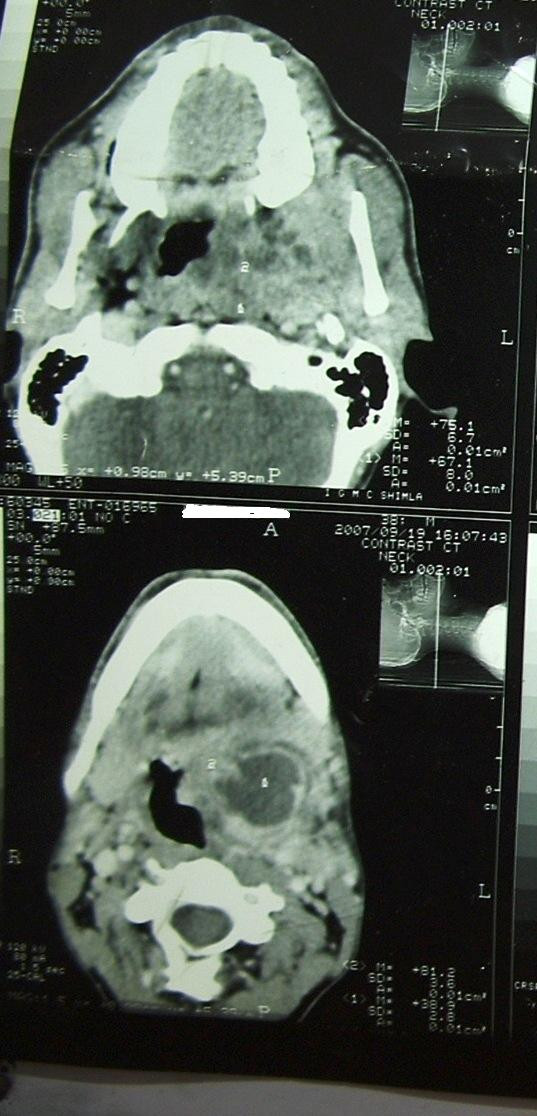
**On 19^th ^Sep 2007, pre-radiotherapy contrast CT scan found heterogeneous enhanced tumor in the nasopharynx with extension up to parapharyngeal area**. The tumor had multiple necrotic areas with largest area in its lower part.

A fine needle aspiration biopsy (FNAB) of the mass was done intra-orally and microscopic examination revealed a poor smear with scanty cells. The pathologist suggested the possibility of a squamous cell carcinoma and advised a direct biopsy for definitive diagnosis. On 19^th ^Sep 2007, computed tomographic (CT) scan of the neck revealed a large mass of heterogeneous density involving the fossa of Rosenmuller, and extending to the valleculla through parapharyngeal space. The mass had heterogeneous contrast enhancement with central necrosis (Fig 1). Based on these features, radiologist suggested a malignant tumor of the nasopharynx.

**Figure 2:**
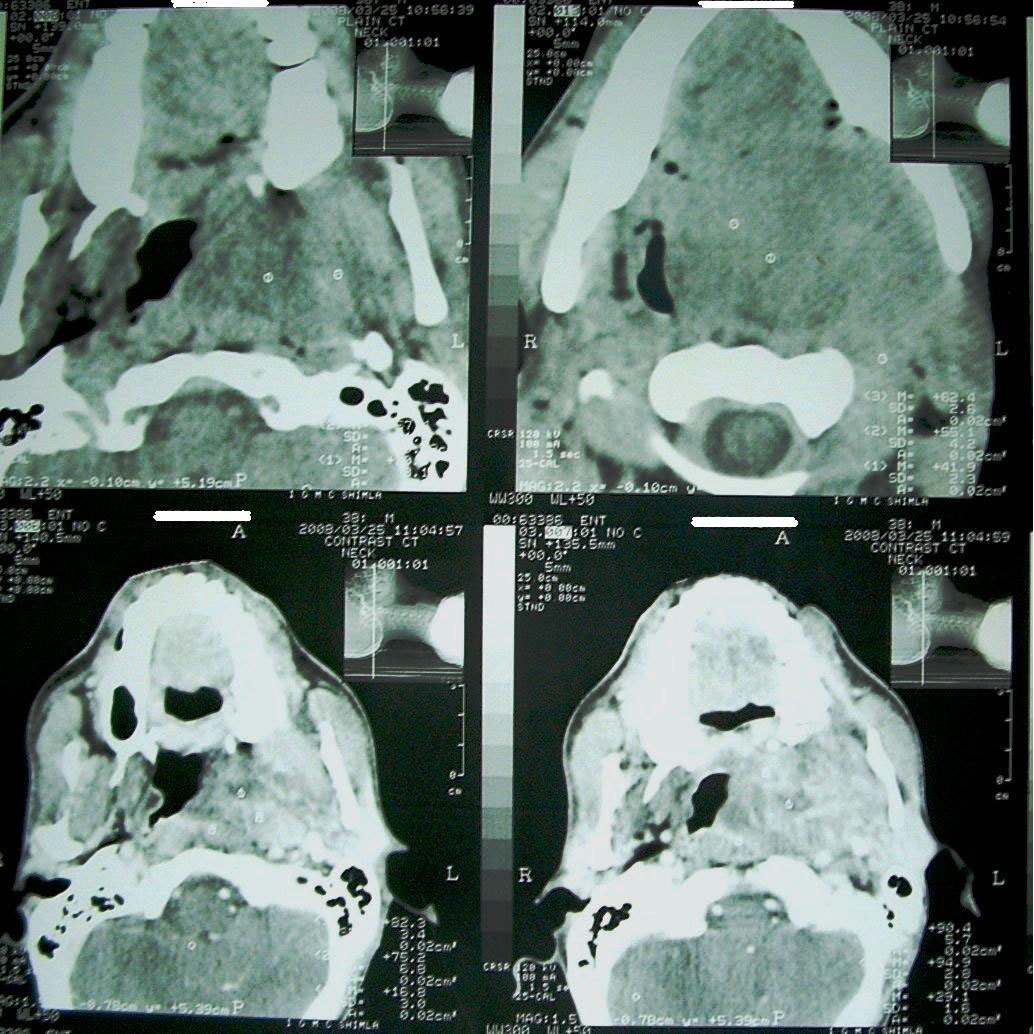
**On 25^th ^March 2008, post-radiotherapy CT scan showed same heterogeneous enhanced tumor of nasopharynx but increased considerably as compared to earlier CT scan (Fig 1)**.

**Figure 3:**
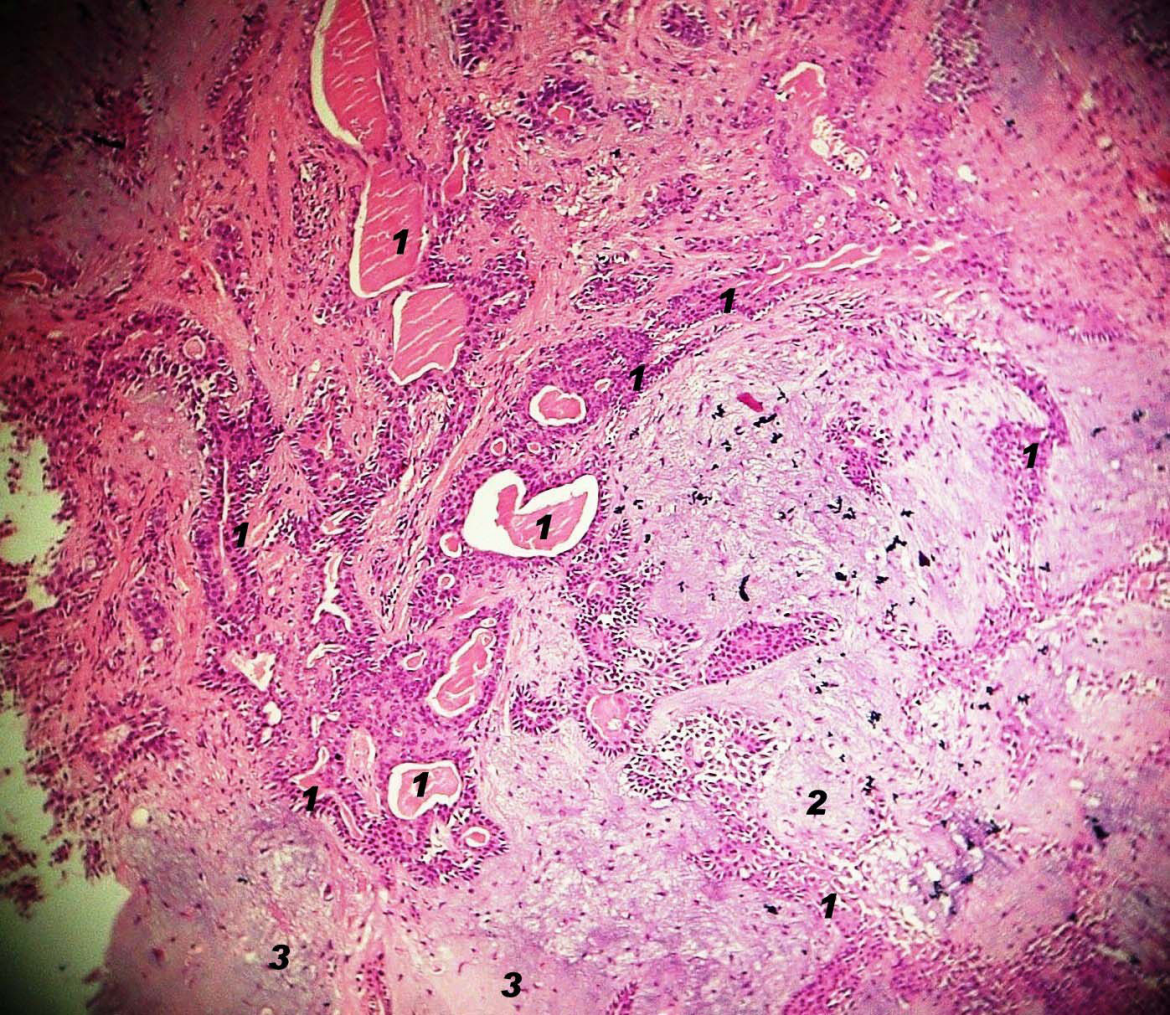
**Photomicrograph of pleomorphic adenoma showing epithelial component (marked as '1') consisting of acini, duct, sheets and cords, and mesenchymal component consisting of myxoid (marked as '2') and chondroid areas (marked as '3')**. (H & E × 100).

A radiation oncologist evaluated the patient and investigations, and began radical chemo-radiation. However, the tumor did not regress despite receiving 20 Gray of radiation, and this led to a suspicion of a benign growth. On 25^th ^March 2008, a fresh CT scan (Fig 2) was done and it showed nasopharyngeal tumor with same features as pre radiotherapy but increased considerably as compared to earlier CT scan. Since there was no histopathological evidence of the carcinoma, the intra-oral incisional biopsy was done and was found to be suggestive of a pleomorphic adenoma. A surgical intervention was planned, and tumor was excised by transpalatal approach under general anesthesia. Histological examination of the excised tumor confirmed a pleomorphic adenoma with no evidence of malignancy (Fig 3). The patient recovered well without any complication and had no recurrence in follow up of 1 year.

## Discussion

In retrospective review of the CT scans, there could be a possibility of tumor arising in the parapharyngeal space and extending to nasopharynx but clinical progression of the tumor and normal neck examination was in favor of nasopharyngeal origin. Parapharyngeal benign tumors have classical features of sub mucosal swelling in the lateral pharyngeal wall with or without extension to retromandibular area or the submandibular triangle, and bimanually ballot ability. These tumors can have additional features of otalgia, neuralgia, trismus, and paresis/palsies of 9^th^, 10^th ^or 11^th ^cranial nerves [1,2].

The fossa of Rosenmuller is a common site of squamous cell carcinoma of the nasopharynx. Nasal obstruction, epistaxis and adenopathy are common symptoms of nasopharyngeal carcinoma; however, the exact diagnosis of nasopharyngeal growth can be made only by an accurate histopathology. The exact site of tumor origin in this case report could be debatable but we present this case because a major mistake occurred by not performing a direct tumor biopsy before starting chemo-radiation.

Palate, nasal cavity and nasopharynx have abundant minor salivary glands and pleomorphic adenoma is the most common benign tumor of these glands. Pleomorphic adenoma arising in the nasal cavity has scanty or absent cartilaginous component which can make histological diagnosis difficult [3]. Compagno and Wong [4] reported that microscopically pleomorphic adenoma of the nasal cavity resembles mixed tumor of the major salivary glands but due to high epithelial cellularity and little stromal component these benign tumor can be mistaken for malignant epithelial neoplasm. Lam *et al*. [5] found skeletal muscle differentiation in pleomorphic adenoma of the nasopharynx which can simulate rhabdomyosarcoma.

Malignant tumors comprise about 40-50% in all the minor salivary gland tumors (7% are carcinoma ex pleomorphic adenoma) [6-9]. Pleomorphic adenoma has three malignant varieties: carcinoma ex pleomorphic adenoma, carcino-sarcoma and metastasizing pleomorphic adenoma. Carcinoma ex pleomorphic adenoma is a rare mixed tumor developing from the epithelial component of the pleomorphic adenoma [9-11]. The malignant transformation in the pleomorphic adenoma has been linked to recurrence and multiple excisions [12,13], laminin, and collagen IV deposition [14,15]. Recently [16], it has been found that loss of β-catenin adhesion molecule is one of the factors responsible for development of pleomorphic adenoma and cytoplasmic accumulation of this molecule causes malignant transformation in pleomorphic adenoma.

CT scan does not provide significant differentiation between carcinoma ex pleomorphic adenoma (CXPA) and benign pleomorphic adenoma as CXPA can have malignant appearance or benign features similar to pleomorphic adenoma. On MRI, low T1 and T2-weighted signal intensity has been suggested as an indicator for carcinoma ex pleomorphic adenoma [17].

An extensive literature review revealed only five cases of pleomorphic adenoma arising in the nasopharynx [5,18-21]. Roh *et al*. [20] excised a pleomorphic adenoma of nasopharynx in a 61-year-old female by endoscope. They advocated use of endoscope in these cases to avoid injury to eustachian tube. Lee *et al*. [21] reported a similar case in an elderly patient who had otalgia, aural fullness and tinnitus. The tumor was excised endoscopically and patient had no recurrence.

**Figure 4:**
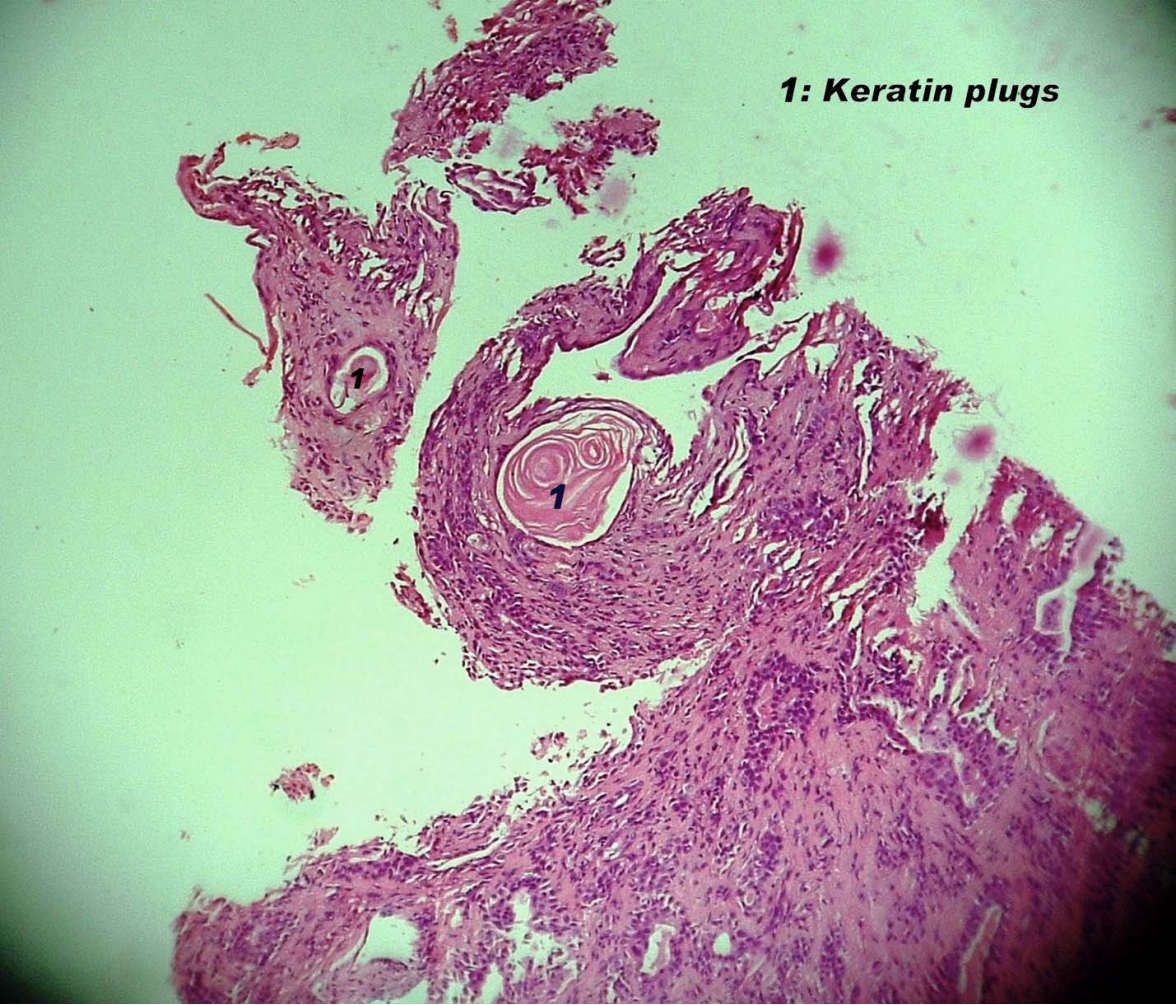
**Photomicrograph of pleomorphic adenoma showing keratin plug of its epithelial component**. (H & E × 100).

Although, radiotherapy is indicated in recurrent pleomorphic adenoma but in our case, diagnosis of carcinoma was made on cytology alone. The patient received chemo-radiation but tumor increased in size, and then tumor was found to be a benign pleomorphic adenoma. Possibly, myoepithelial or epithelial atypia with keratin debris in pleomorphic adenoma (Fig 4) led to cytological diagnosis of carcinoma. To make the issue more complex, there could be a possibility that the adenoma was undergoing malignant transformation and these changes were picked up in the cytology. After radiotherapy, malignant part disappeared and consequently we did not find any evidence of malignancy in the excised pleomorphic adenoma.

## Conclusion

The pleomorphic adenoma was undergoing malignant changes or it was a misdiagnosis, we could not ascertain the exact pathology, as we had no pre-radiotherapy histopathological evidence. However, who was at fault: clinician by not following the advice of pathologist for a direct biopsy, or oncologist who started chemo-radiation without any histopathological proof? We cannot blame anyone rather than ourselves as the clinician are the most accountable.

In this case report, many questions remained unanswered but a lesson learnt was:

1. The possibility of benign tumor should be kept in the differential diagnosis of nasopharyngeal tumor without adenopathy.

2. Clinician should follow pathologist's advice, carefully interpret the cytological diagnosis, and consult the pathologist, whenever in doubt.

3. An adequate tissue specimen should be obtained especially for cytological diagnosis as pleomorphic adenoma can simulate as a carcinoma.

4. No patient should be taken for chemo-radiation without any histopathological evidence.

## Consent

Written informed consent was obtained from the patient for publication of this case report and any accompanying images. A copy of the written consent is available for review by the Editor-in-Chief of this journal.

## List of abbreviations used

**FNAB: **Fine needle aspiration biopsy; **CT: **Computed tomography; **MRI: **Magnetic resonance image; **CXPA: **Carcinoma ex pleomorphic adenoma; **H & E: **Hemotoxylin and Eosin

## Financial and Non-Financial Disclosure

None

## Competing interests

The authors declare that they have no competing interests.

## Authors' contributions

JST was the principal investigator and involved with management of the patient, design, concept and writing of the paper. He takes the responsibility for the integrity of the article. NKM was involved with management of the case, revision and final approval of the paper. SM was responsible for the histopathological analysis, critical revision and final approval of the paper. DRS was involved with management of the case, revision and final approval of the paper. AT was involved in design, literature review, data collection, drafting and final approval of the paper.

## Pre-publication history

The pre-publication history for this paper can be accessed here:

http://www.biomedcentral.com/1472-6815/10/2/prepub

## References

[B1] Carrau RL Myers EN Johnson JT Management of tumours arising in the parapharyngeal space Laryngoscope 1990 100 583 589 10.1288/00005537-199006000-00006 2348735

[B2] Hughes KV Oslen KD McCaffery TV Parapharyngeal space neoplasms Head Neck 1995 17 124 130 10.1002/hed.2880170209 7558809

[B3] Heffner DK Classification of human upper respiratory tract tumors Environ Health Perspect 1990 85 219 29 10.2307/3430685 2384062 PMC1568346

[B4] Campagno J Wong RT Intranasal mixed tumors (pleomorphic adenomas): a clinicopathological study of 40 cases Am J Clin Pathol 1977 68 2 213 8 195456 10.1093/ajcp/68.2.213

[B5] Lam PWY Chan JKC Sin VC Nasal pleomorphic adenoma with skeletal muscle differentiation: Potential misdiagnosis as rhabdomyosarcoma Hum Pathol 1997 28 11 1299 1302 10.1016/S0046-8177(97)90205-7 9385937

[B6] Beckhardt RN Weber RS Zane R Garden AS Wolf P Carrillo R Luna MA Minor salivary gland tumors of the palate; clinical and pathologic correlates of outcome Laryngoscope 1995 105 1155 60 10.1288/00005537-199511000-00003 7475867

[B7] Neely MN Rohrer MD Young SK Tumors of minor salivary glands and the analysis of 106 cases J Okla Dent Assoc 1996 86 50 2 9540689

[B8] Waldron CA el-Mofty SK Gnepp DR Tumors of the intraoral minor salivary glands; a demographic and histologic study of 426 cases Oral Surg Oral Med Oral Pathol 1988 66 323 33 10.1016/0030-4220(88)90240-X 2845326

[B9] Furukawa M Suzuki H Matsuura K Takahashi E Suzuki H Tezuka F Carcinoma ex pleomorphic adenoma of the palatal minor salivary gland with extension into the nasopharynx Auris Nasus Larynx 2001 28 279 81 10.1016/S0385-8146(01)00052-9 11489377

[B10] Gnepp DR Malignant mixed tumors of the salivary glands: a review Pathol Annu 1993 28 279 328 8380049

[B11] Akan H Yildiz L Unal R Carcinoma ex pleomorphic adenoma of minor salivary gland with pulmonary metastasis Diagn Interv Radiol 2008 14 3 5 18306136

[B12] Spiro RH Koss LG Hajdu SI Strong EW Tumors of minor salivary origin. A clinicopathologic study of 492 cases Cancer 1973 31 117 29 10.1002/1097-0142(197301)31:1<117::AID-CNCR2820310116>3.0.CO;2-7 4345606

[B13] Livolsi VA Perzin KH Malignant mixed tumors in salivary glands; carcinomas arising in benign mixed tumors, a clinicopathologic study Cancer 1977 39 2209 30 10.1002/1097-0142(197705)39:5<2209::AID-CNCR2820390540>3.0.CO;2-8 192443

[B14] Karja V Syrjanen K Syrjanen S Collagen IV and tenascin immuno-reactivity as prognostic determinant in benign and malignant salivary gland tumors Acta Otolaryngol 1995 115 569 75 10.3109/00016489509139369 7572138

[B15] Felix AF Costa Rosa J Fonseca I Cidadao A Soares J Laminin and collagen IV in pleomorphic adenoma and carcinoma ex pleomorphic adenoma: an immuno-histochemical study Hum Pathol 1999 30 964 9 10.1016/S0046-8177(99)90251-4 10452510

[B16] do Prado RF Consolaro A Taveira LAA Expression of β-catenin in carcinoma in pleomorphic adenoma, pleomorphic adenoma and normal salivary gland: An immunohistochemical study Med Oral Patol Oral Cir Bucal 2006 11 E247 51 16648762

[B17] Som PM Brandwein MS Som PM, Curtin HD Salivary glands Head and Neck Imaging 2008 14 St Louis: Mosby 3 5 2003: 2070. Retraction in: Akan H, Yildiz L,Unal R. Carcinoma ex pleomorphic adenoma of minor salivary gland with pulmonary metastasis. Diagn Interv Radiol.

[B18] Li TS Minor salivary gland tumors of the nasopharynx [Abstract] Zhonghua Zhong Liu Za Zhi 1990 12 2 127 9 2169388

[B19] Amilibia E Nogues J Sandoval M Arias G Dicenta M Minor salivary gland tumor in nasopharynx [Abstract] Acta Otorrhinolaringol Esp 1997 48 8 671 3 9528140

[B20] Roh JL Jung BJ Rha KS Park CI Endoscopic resection of pleomorphic adenoma arising in the nasopharynx Acta Otolaryngol 2005 125 8 910 12 10.1080/00016480510039003 16158542

[B21] Lee SL Lee CY Silver SM Kuhar S Nasopharyngeal pleomorphic adenoma in the adult Laryngoscope 2006 116 7 1281 3 10.1097/01.mlg.0000221972.07176.38 16826077

